# ABO Blood Groups as a Disease Marker to Predict Atrial Fibrillation Recurrence after Catheter Ablation

**DOI:** 10.3390/jpm13020355

**Published:** 2023-02-17

**Authors:** Shin-Huei Liu, Chheng Chhay, Yu-Feng Hu, Yenn-Jiang Lin, Shih-Lin Chang, Li-Wei Lo, Fa-Po Chung, Ta-Chuan Tuan, Tze-Fan Chao, Jo-Nan Liao, Chin-Yu Lin, Ting-Yung Chang, Ling Kuo, Chih-Min Liu, An Nu-Khanh Ton, Dony Yugo, Shih-Ann Chen

**Affiliations:** 1 Heart Rhythm Center, Division of Cardiology, Department of Medicine, Taipei Veterans General Hospital, Taipei 11220, Taiwan; 2 Division of Holistic and Multidisciplinary Medicine, Department of Medicine, Taipei Veterans General Hospital, Taipei 11217, Taiwan; 3 Institute of Clinical Medicine and Cardiovascular Research Center, National Yang Ming Chiao Tung University, Taipei 11217, Taiwan; 4 Faculty of Medicine, School of Medicine, National Yang Ming Chiao Tung University, Taipei 11217, Taiwan; 5 Cardiovascular Center, Taichung Veterans General Hospital, Taichung 407219, Taiwan

**Keywords:** ABO blood group, atrial fibrillation, late recurrence, very late recurrence, biomarker

## Abstract

Chronic inflammation harbors a vulnerable substrate for atrial fibrillation (AF) recurrence after catheter ablation. However, whether the ABO blood types are associated with AF recurrence after catheter ablation is unknown. A total of 2106 AF patients (1552 men, 554 women) who underwent catheter ablation were enrolled retrospectively. The patients were separated into two groups according to the ABO blood types, the O-type (n = 910, 43.21%) and the non-O-type groups (A, B, or AB type) (n = 1196, 56.79%). The clinical characteristics, AF recurrence, and risk predictors were investigated. The non-O type blood group had a higher incidence of diabetes mellitus (11.90 vs. 9.03%, *p* = 0.035), larger left atrial diameters (39.43 ± 6.74 vs. 38.20 ± 6.47, *p* = 0.007), and decreased left ventricular ejection fractions (56.01 ± 7.33 vs. 58.65 ± 6.34, *p* = 0.044) than the O-type blood group. In the non-paroxysmal AF (non-PAF) patients, the non-O-type blood groups have significantly higher incidences of very late recurrence (67.46 vs. 32.54%, *p* = 0.045) than those in the O-type blood group. The multivariate analysis revealed the non-O blood group (odd ratio 1.40, *p* = 0.022) and amiodarone (odd ratio 1.44, *p* = 0.013) were independent predictors for very late recurrence in the non-PAF patients after catheter ablation, which could be applied as a useful disease marker. This work highlighted the potential link between the ABO blood types and inflammatory activities that contribute to the pathogenic development of AF. The presence of surface antigens on cardiomyocytes or blood cells in patients with different ABO blood types will have an impactful role in risk stratification for AF prognosis after catheter ablation. Further prospective studies are warranted to prove the translational benefits of the ABO blood types for the patients receiving catheter ablation.

## 1. Introduction

Atrial fibrillation (AF) is a common global health issue in the aging society. Not only causing poor quality of life, but the AF also increases adverse cardiovascular (CV) events, including death, heart failure (HF), and stroke. The present therapeutic guideline recommended the importance of stroke prevention, rhythm, and rate control are considered the cornerstone of a standardized AF treatment [1]. Early initiation of rhythm control attenuates the mounting risk of HF or stroke. It improves the long-term prognosis for AF patients diagnosed within one year of first onset [2]. The attempt to restore sinus rhythm and decrease AF burden is a crucial strategy to prevent CV consequences of HF; however, it has a marginal effect on the prevention of stroke [2]. Strategies for rhythm control included pharmacological approaches through antiarrhythmic drugs (AAD) or nonpharmacological approaches through catheter ablation [1]. The emergence of catheter ablation for AF patients contributed to a high success rate of approximately 80% after the first procedure of pulmonary vein isolation (PVI) [1,2]. Therefore, catheter ablation was recommended as the class I indication for symptomatic drug-refractory AF patients in the current guideline [2,3].

Catheter ablation reduces the incidence of stroke and all-cause mortality in selected patients compared with AADs [1]. However, approximately 20% of AF patients experience recurrence within one year of catheter ablation [1,4]. Identifying the risk for recurrence is important in patients receiving catheter ablation for shared decision-making before the procedure. This also helps the physician to propose a rigorous follow-up for the recurrence of arrhythmias after the procedure. The risk factors that contribute to AF recurrence after catheter ablation include AF duration, AF types, pre-procedural left atrial diameter (LAD), hypertension (HTN), stroke, and cardiac structural diseases [1]. Various models of risk factors have been developed to predict AF recurrence after catheter ablation. The search for risk factors that aid clinical decisions and early detection of AF recurrence is vital. However, the predictive models for 1-year AF recurrence now remain unsatisfactory and heterogeneous in a meta-analysis of 27 studies [5,6]. Early detection of AF recurrence is vital for clinical decisions. More comprehensive risk factors to increase predictive power for recurrence remain an ongoing study field for the clinical management of patients. The serum biomarkers have been extensively studied and included as the risk factor to predict AF recurrence after catheter ablation [7]. Among various biomarkers, fibrotic and inflammatory biomarkers have a convincing link to AF pathophysiology and high predictive power for AF recurrence. The underlying mechanisms of AF stem from the interaction of overactivated fibrotic and inflammatory cascades. Cardiac fibrosis harbors myocardial substrate that potentially contributes to AF formation and triggers [7]. Fibrotic biomarkers such as transforming growth factor-beta (TGF-β), matrix-metalloproteinase (MMP), or galectin-3 (Gal-3) were associated with catheter ablation outcomes in AF patients [7]. Higher serum levels of fibrotic biomarkers are associated with AF recurrence after catheter ablation [7]. Inflammatory biomarkers such as C-reactive protein (CRP), tumor necrosis factor-alpha (TNF-α), or interleukin-6 (IL-6) were also higher in AF patients with recurrence after catheter ablation [7]. Despite mounting evidence supporting the critical link between inflammation and AF recurrence, some studies suggest the increased levels of these inflammatory biomarkers simply represent the signs of local inflammation and myocardial injury after catheter ablation and are not as valuable as a predictor of AF recurrence [8]. Numerous studies demonstrated the association of ABO blood groups with inflammation, thromboembolism, aging diseases, and CV diseases [9]. Patients with non-O-type blood groups (A, B, or AB type) have a higher risk of thromboembolic events such as coronary artery diseases, cerebrovascular ischemic events, and peripheral vascular disease than those with the O-type blood group [10]. 

To be especially noted in patients with atrial fibrillation (AF), the non-O-type blood groups (A, B, or AB type) were associated with active inflammatory status, as reflected by increased levels of von Willebrand factors (VWF) and inflammatory factors. As cardiac inflammation underlies AF pathogenesis by the induction of atrial electrical and structural remodeling, increases in AF vulnerability or recurrence after catheter ablation in patients with increased cardiac inflammation are observed [11]. The inflammatory biomarkers have been applied as clinical surrogates to predict AF attack and recurrence after catheter ablation. The close link of ABO blood group antigens to inflammation raised an interesting hypothesis that the blood group might be considered a surrogate marker for cardiac inflammation to predict AF attack [11].

Catheter ablation is a standard therapy for AF patients to maintain sinus rhythm. The AF recurrence at one year remains approximately 20% of AF patients receiving catheter ablation. Developing biomarkers to predict AF recurrence is crucial, which helps the physician identify the patients at risk for AF recurrence. Therefore, the therapies could be adjusted, in the early stages, and the patient could be followed up more closely. In the present work, we aimed to investigate whether ABO blood groups could be used as a biomarker for the prediction of arrhythmia recurrence in AF patients receiving catheter ablation in the single-center retrospective cohort.

## 2. Material and Methods

### 2.1. Study Population and Hospital Database

All symptomatic drug-refractory AF patients who underwent catheter ablation (January 2004 to January 2018) were enrolled at Taipei Veterans General Hospital, Taiwan. The AF diagnosis and documentation were guided by the 9th version of the International Classification of Diseases (ICD) and Clinical Modification (ICD-9-CM) codes. The ICD codes were registered by cardiologists in charge of the patient’s diagnosis and treatment. Any patient who presented with a bed-ridden status, pregnancy, hemodynamic unstable condition, intracardiac thrombus, younger than 18 years old, older than 80 years old were excluded from this investigation. All AF patients were further divided into 2 groups (O-type blood group and non-O-type blood groups). This study included the baseline characteristics of personal history, AF types, blood group, age, gender, CHA_2_DS_2_-VASc score, underlying comorbidities, 12-lead electrocardiogram (ECG), AAD, echocardiogram, and AF recurrence were collected from the hospital’s medical system. The risk factors of AF recurrence including patient’s age, HTN, diabetes mellitus (DM), HF, dyslipidemia, and LAD were also investigated. This study protocol was reviewed and approved by the Ethics of Institutional Review Board of the Taipei Veterans General Hospital, Taiwan (IRB: 2021-01-026CC).

The AF-related symptoms included fatigue, dizziness, palpitations, dyspnea, chest pain, and anxiety. The European Heart Rhythm Association (EHRA) symptom score was applied by the physician to identify and quantify AF-related symptoms for guidance in a symptom-driven treatment approach [3]. The AF-related symptoms are defined from mild to disabling symptoms according to their adverse impact on regular activity. The recommendation of catheter ablation for drug-refractory AF patients was in concordance with the latest AF guideline when patients failed or were intolerant of class I or III AADs to improve AF symptoms [3]. In some patients with recurrent symptomatic paroxysmal AF, we used catheter ablation as a reasonable initial rhythm-control strategy after weighing the risks and outcomes of drug and ablation therapy and sharing decision-making with the patients.

According to the latest AF guideline, paroxysmal AF (PAF) was defined as AF that terminated spontaneously or terminated with intervention within 7 days of onset [3]. Persistent AF was defined as AF that continuously sustained beyond 7 days, including episodes terminated by cardioversion (drugs or electrical cardioversion) after ≥7 days [3]. Long-standing persistent AF was defined as continuous AF of >12 months when deciding to adopt a rhythm control strategy [3]. In the present work, persistent AF and long-standing persistent AF were defined as non-PAF.

### 2.2. Catheter Ablation Procedure

According to our protocol, symptomatic drug-refractory AF patients discontinued their AADs for >5 half-lives before the procedure. Amiodarone was stopped 2 weeks before the procedure because of the long half-life. Patients all received adequate oral anticoagulant before catheter ablation according to the guideline [3]. During the procedure, standard cardiac electrophysiology (EP) study, electroanatomic map (EAM), signal analysis, identification of pulmonary vein (PV) and the non-PV beats, and PV isolations (PVI) were performed [12]. The patients underwent either local anesthesia or general anesthesia using propofol for induction (1–2 mg/kg) followed by sevoflurane inhalation for maintenance performed by an anesthesiologist [12]. During the procedure, intracardiac signals of electrograms (EGM)were monitored by our Lab System Pro (BARD, Boston Scientific, Lowell, MA, USA) through a signal filter at 30–300 Hz. [12] First, all patients underwent 3D electroanatomical mapping (EAM) using the CARTO 3 system (Biosense Webster, Irvine, CA, USA) or the Ensite system (Abbott, Abbott Park, IL, USA) during sinus rhythm or atrial arrhythmias. Atrial voltage and isochronal maps were constructed during sinus rhythm. For patients with a sinus rhythm, a programmed electrical atrial pacing was applied among sinus rhythm patients to induce atrial arrhythmias and identify trigger foci. Additional isoproterenol (2–6 μg/min) was administered for sinus rhythm patients who cannot develop atrial arrhythmias under atrial pacing [12].

The catheter ablations were performed by an anatomic-based linear ablation approach at the PV antrum. The ablation lesions were achieved through a point-by-point approach using ablation catheters based on the selection of the EAM systems [12]. Successful PVI was demonstrated by confirming the entrance and exit blocks of the PVs. In the patients with PAF, after completion of the circumferential lesion set, the ipsilateral superior and inferior PVs were mapped carefully by a circular catheter recording (Spiral SC, AF Division, St. Jude Medical, Inc., Little Canada, MN, USA). Finally, an atrial inducibility test was performed at the end of the procedure [12].

In patients with non-PAF, substrate modification (linear ablation or complex fractionated atrial electrogram ablation [CFAE]) was performed if the AF did not terminate after PVIs. The endpoint was a prolongation of the cycle length, elimination of CFAEs, or abolishment of local fractionated potentials. Linear ablation included roof or mitral lines in the left atrium (LA) [12]. Linear ablation was not performed if there were no clinically relevant atrial arrhythmias, such as atrial flutters. If atrial arrhythmia persisted after the PVIs and substrate modifications, sinus rhythm was restored by electrical cardioversion. The ablation endpoint was a restoration of sinus rhythm during the procedure. If the AF could not be terminated after substrate modification, electrical cardioversion was performed to restore sinus rhythm [12].

### 2.3. Outpatient Follow-Up and AF Recurrence

After catheter ablation, all patients received AADs for at least 6–8 weeks to prevent acute recurrence during the blanking period. Acute recurrence after catheter ablation is defined as the attack of atrial arrhythmias within three months of the blanking period [13]. A blanking period of three months is employed after ablation when reporting efficacy outcomes [13]. Acute inflammation after catheter ablation leads to a potential arrhythmogenic effect in approximately 35–40% of post-ablation AF patients [3,13]. Acute recurrence within the blanking period is unrelated to long-term AF recurrence because inflammation usually subsides after the blanking period [3,13]. Any atrial arrhythmias within 3 months after the catheter ablation (the blanking period) were considered transient post-procedural arrhythmias that attenuated through time and were not classified as treatment failure [3]. The definition of AF recurrence was the presence of symptomatic or asymptomatic sustained atrial arrhythmias (>30 s) after the blanking period identified on the 12-lead ECG, 24-h Holter recorder, or 1-week event recorder. The AF recurrence was further classified into 2 categories according to recurrence time as late recurrence (3 months to 1 year after catheter ablation) and very late recurrence (>1 year after catheter ablation) [3].

All patients received a series of outpatient follow-ups 1–2 weeks, 1 month, and every 3 months after catheter ablation. One year after the catheter ablation, the outpatient follow-ups were changed to every 6 months. All patients received a resting 12-lead ECG during every outpatient follow-up. The 12-lead ECGs were performed by trained cardiology technicians with a 30-s observation on the 12-lead ECG monitor to determine whether the atrial arrhythmias were sustained [3]. A 24-h Holter recorder or a 1-week event ECG recorder was regularly arranged for patients during each follow-up or those who had negative findings on a 12-lead ECG but were presented with symptoms of AF. Symptoms of AF included frequent palpitation, lightheadedness, dizziness, chest tightness, shortness of breath, exercise intolerance, or general weakness. All 12-lead ECGs, 24-h Holter recordings, and 1-week event ECG recorders were performed and analyzed by trained cardiology technicians. All interpretations and final diagnoses of 12-lead ECGs, 24-h Holter recorders, and 1-week event ECG recorders were performed by board-certified cardiologists during outpatient follow-ups. If an AF recurrence on a 12-lead ECG, 24-h Holter recorder, or 1-week event ECG recorder was confirmed by the cardiologist, AADs might be prescribed to prevent AF recurrence. In addition to routine outpatient follow-ups, patients who experienced symptoms of AF recurrence outside the hospital were strongly encouraged to visit an earlier outpatient follow-up. The AF recurrence symptoms included fatigue, dizziness, palpitations, dyspnea, chest pain, and anxiety. Finally, all patients received an echocardiogram before and after catheter ablation for investigation.

Therapeutic strategies that improve long-term rhythm control were recommended and educated, including weight loss, strict control of chronic diseases, and avoiding AF-related triggers. Chronic diseases included HTN, DM, HF, and dyslipidemia. The AF-related triggers included alcohol drinking or smoking [3]. The importance of identifying AF recurrence at an early stage is associated with a patient’s quality of life and long-term recovery. The AF patients who neglect the presence of an AF recurrence after catheter ablation could potentially suffer from AF-related symptoms leading to poor quality of life. Among the symptoms of AF, dizziness and lightheadedness could potentially cause unnecessary traumatic injuries. The underlying mechanism of AF recurrence during the blanking period suggested a potential acute local inflammation at the catheter ablation region that required anti-inflammatory agents and additional AADs for rhythm control [3]. Therefore, an earlier outpatient follow-up with appropriate adjustment of AADs for these patients could minimize complications derived from AF recurrence [3].

### 2.4. Statistical Analyses

All analyses were calculated with the IBM SPSS Statistics 22 (SPSS Inc., Chicago, IL, USA). Continuous variables were expressed as mean ± standard deviation, and categorical variables as counts (percentages). The two-sample independent T-test was used to analyze continuous variables. Categorical data were compared using a Chi-square test with Yates correction or the Fisher exact test. Logistic regression analysis was used for identifying the independent predictor of very late AF recurrence. A *p*-value of less than 0.05 was considered significant.

## 3. Results

### 3.1. Baseline Characteristics

A total of 2106 patients (non-O-type blood groups, *n* = 1196, 56.8%) were enrolled. Table 1 shows the comparison of patient characteristics between the O-type and the non-O-type blood groups. No significant differences were found in age, gender, hypertension (HTN), dyslipidemia, AF types, smoking, and AADs between the two groups (Table 1). Patients in the non-O-type blood groups have higher incidences of diabetes mellitus (DM) but lower alcohol consumption than those in the O-type blood group. In the non-O-type blood groups, the LAD was significantly larger than those in the O-type blood group, whereas the left ventricular ejection fraction (LVEF) was significantly decreased than those in the O-type blood group [11].

### 3.2. Predictors of AF Recurrence

**Figure 1:**
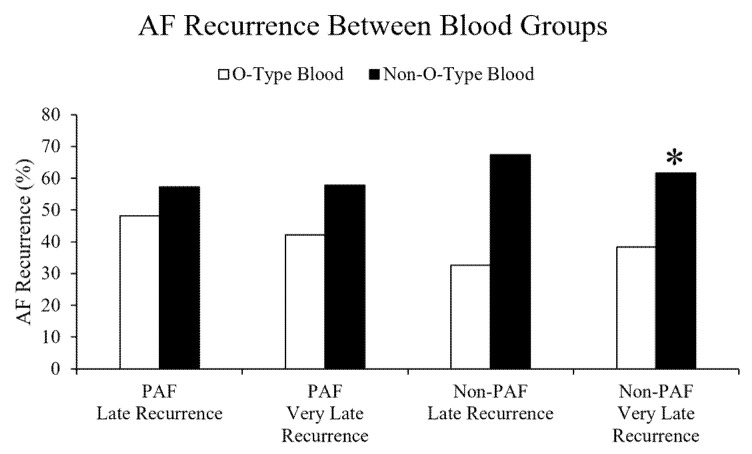
Incidence of AF recurrence between blood groups after catheter ablation. The incidence of late and very late AF recurrence in the PAF patients revealed no difference between the different blood groups. The very late AF recurrence of the non-O-type blood groups (A, B, or AB type) in the non-PAF patients was significantly higher than those in the O-type blood group. * *p* < 0.05 compared with the O-type blood group within the category.

The AF recurrence was analyzed according to AF classifications (PAF and non-PAF). In PAF patients after catheter ablation, there were no differences in late and very late recurrences between the O-type and the non-O-type blood groups, respectively (Figure 1).

In the non-PAF patients after catheter ablation, the very late recurrence of the non-O-type blood groups was significantly higher than those in the O-type blood group (Figure 1). Similarly, a marginally higher late recurrence was observed in the patients with the non-O-type blood groups than in the O-type blood group. Table 2 demonstrates the comparison of patient characteristics between no recurrence and very late recurrence in non-PAF patients after catheter ablation. Patient characteristics of age, gender, HTN, DM, dyslipidemia, CHA_2_DS_2_-VASc score, AADs, alcohol consumption, smoking, and echocardiogram parameters showed no difference between the no recurrence and very late recurrence groups (Table 2). Compared to those without recurrence, more patients with very late recurrence received AADs, including propafenone (32.55% vs. 2.67%) or amiodarone (66.33% vs. 12.24%). The patients with very late recurrence also had a marginally higher CHA_2_DS_2_-VASc Score than those without recurrence. The univariate and multivariate analysis revealed that the non-O blood group (odds ratio:1.40, 95% CI: 1.05–1.86) and amiodarone (odds ratio: 1.44, 95% CI: 1.08–1.93) were independent predictors for very late recurrence in the non-PAF patients after catheter ablation (Table 3).

## 4. Discussion

### 4.1. Main Findings

The non-PAF patients with non-O-type blood groups (A, B, or AB type) have higher incidences of very late AF recurrence after catheter ablation. The non-O type blood group was an independent predictor for AF recurrence. This suggests that different ABO blood groups could be applied as a biomarker for risk stratification of AF recurrence in patients after catheter ablation.

### 4.2. ABO Blood Groups as Biomarker for AF Recurrence

Accurate blood group typing is critical before a blood transfusion. This information is considered essential for all the patients before catheter ablation of AF because blood transfusion is sometimes unfortunately needed for bleeding during the procedure. However, this test is rarely applied as an alternative application. In the present work, we showed that the ABO blood groups were an independent predictor of very late AF recurrence in the non-PAF patients. This extends the potential application of the ABO blood group from blood transfusion to a clinical biomarker to predict AF recurrence. In addition to the conventional risk factors, including age, gender, chronic diseases, non-pulmonary vein foci, and AF persistence, the non-O-type blood groups (A, B, or AB type) are a simple and effective biomarker with an independent predictive power for AF recurrence [14,15]. The late and very late recurrence of the non-O-type blood groups (A, B, or AB type) was higher than those in the O-type blood group in the non-PAF. Therefore, in patients with blood type A or B, either the catheter ablation procedure for AF or the clinical follow-up after ablation should be more cautiously performed to avoid negligence for AF recurrence.

### 4.3. Clinical Applications of ABO Blood Groups in AF Patients Receiving Catheter Ablation

The non-O-type blood groups (A, B, or AB type) are an independent risk factor for AF recurrence. Several potential clinical applications of ABO blood groups could be considered in the future. First, the risk prediction model, including ABO blood groups, will help doctors set up a treatment plan and make shared decision-making. This includes the explanation of recurrent risk and post-procedure patient care. Moreover, the patients and families will be informed of their relevant risky condition after the procedure. As these patients have a higher risk for AF recurrence, some relevant symptoms related to AF recurrence could be explained thoroughly to avoid any negligence of arrhythmia attack. The AF patients who neglect the presence of an AF recurrence after catheter ablation could potentially suffer from AF-related symptoms and have a poor quality of life. Early detection of AF recurrence is not only associated with improved quality of life but also changes the therapeutic plan. Following a 3-month blanking period, it is reasonable to incorporate an additional 1–3 months of therapy for consolidation. The adjustment of AADs and/or electrical cardioversion can be performed if AF recurrence could be detected as early as possible. The appropriate treatment for these patients might mitigate complications from AF recurrence [3]. The initiation of the consolidating or rescuing therapy also might lead to better long-term recovery and CV outcomes.

Second, the patients after catheter ablation are usually followed regularly after catheter ablation by cardiac monitoring devices. In order to detect AF recurrence in the early stages, the non-O-type blood groups (A, B, or AB type) might need a more frequent and longer duration of arrhythmia monitoring. The typical monitoring duration of the non-invasive recorders ranges from 24 h to months. As some of the attacks are minimally symptomatic, patch monitoring for 1–3 weeks, rather than 24-h Holter recording might be considered if AF recurrence is suspected in the non-O-type blood groups (A, B, or AB type).

Third, AF recurrence is related to the reconnection of PVs or the triggers from non-PV areas. In the patients with the non-O-type blood groups (A, B, or AB type), a more intensive induction of arrhythmic triggers might be considered. The PV reconnection could be checked cautiously by PV exit pacing after catheter ablation at the end of the procedure. Although it remains controversial, the adenosine test can be used to unmask dormant conduction after PVI, ensure persistent bidirectional PV conduction block, and possibly prevent future reconnection.

Finally, anti-inflammatory therapy might be used for patients with non-O-type blood groups (A, B, or AB type). This is an interesting hypothesis and needs forthcoming solid clinical evidence to prove its feasibility. However, anti-inflammatory therapy is not a new story for AF. For example, colchicine, statins, steroid, or non-steroid anti-inflammatory drugs have been reported to decrease AF recurrence in small cohorts.

### 4.4. Relationship between ABO Blood Groups and Inflammation

The United Biobank investigation suggests that the ABO genotypes are strongly associated with common inflammatory and CV diseases. In addition, the ABO blood groups are associated with different levels of VWF and platelet endothelial cell adhesion molecule-1 [9].

In a genome-wide association study that investigated patients with the acute coronary syndrome, genetic variants at the ABO blood group locus were associated with interleukin-10 levels [16]. Furthermore, the O-type surface antigen was also associated with an increased risk of CV death, all causes of death, and recurrent myocardial infarction [16]. The crucial link between interleukin-10 and ABO blood groups might explain the impact of the ABO blood groups on clinical outcomes, as inflammatory cascades are critically involved in the pathogenesis of coronary artery disease. The association between blood groups and circulating inflammation-related molecules was also reported in the analysis of the Prostate, Lung, Colorectal, and Ovarian Cancer Screening Trial, including 3537 non-Hispanic white participants. ABO blood type was associated with levels of soluble vascular endothelial growth factor receptor 2 (sVEGFR2), sVEGFR3, and soluble glycoprotein 130 (sGP130) [17]. These pieces of clinical evidence suggest the potential pathophysiological links of the ABO blood groups to CV diseases, and the mechanisms are attributed to the over-activated inflammatory pathways.

The ABO blood groups are determined by antigen modifications on glycoproteins and glycolipids. The expressions of the ABO blood group antigens are found on the cardiomyocytes of the endocardium [18]. Different ABO antigens (type A or B) might activate plasma inflammatory cytokines and anticoagulation factors and are linked to localized myocardial injury. The genome-wide association studies suggested that the ABO blood groups are associated with different inflammatory profiles among CV diseases, including VWF, tissue factor, thrombin, thrombomodulin, interleukin −6, interleukin −10, tumor necrosis factor -α, histamine, bradykinin, and prostaglandin [19]. All these biomarkers promote the migration of leukocytes into the tissue and the inflammatory cascade [19].

Among these inflammatory biomarkers, the VWF level might be the most important factor that links the ABO blood groups and AF recurrence. The human ABO blood group led to different plasma levels of VWF and factor VIII (FVIII), which contributed to coagulopathy and CV diseases [20,21]. Patients with the type A or B blood group have plasma VWF and FVIII levels approximately 25% higher than patients with the O-type blood group indicating the link between the non-O-type blood groups (A, B, or AB type) and coagulation factors. In the non-O-type blood groups (A, B, or AB type) patients, blood group antigens are expressed on the oligosaccharide side chains of VWF molecules, causing the slow clearance of VWF levels [22]. Increased VWF expression on the atrial endocardium in AF patients increases atrial structural remodeling and predicts AF recurrence in patients receiving surgical AF ablation [23,24]. Overall, the ABO blood groups were potentially linked to atrial inflammation and remodeling. The ABO blood groups might change the activation of the local or systemic inflammation, endothelial dysfunction, platelet aggregation, and coagulation, which all play critical roles in the pathogenesis of AF [25]. Through similar mechanisms, the surface antigen for the ABO blood groups potentially participates in the inflammation activities, which are involved in the disease process and AF prognosis [17]. This explains the possible mechanisms to predict AF recurrence and the differential prediction power of blood groups between the PAF and non-PAF. In the patients with non-PAF, atrial substrate remodeling is prominent and much worse than in those s with PAF. Therefore, the ABO blood groups could potentially predict AF recurrence in patients with non-PAF.

## 5. Limitations

The present study has several limitations. First, this study was a retrospective design within a single medical center, therefore, it cannot represent the general population. A prospective multicenter randomized controlled trial might be needed to clarify our findings. Second, the catheter ablation procedural variables or ablation strategies that could affect long-term outcomes still needs to be thoroughly investigated. Different strategies between operators and operating systems might be related to procedure outcomes, although the procedure endpoints have been standardized in our center. Third, the influence of drug-drug interaction in underlying chronic diseases needed to be thoroughly investigated. The follow-up of the patients in the present retrospective cohort is not as rigorous as those in the clinical trials. The monitoring devices for post-procedure arrhythmia monitoring, as physicians decided, could change the monitoring durations of arrhythmias. Finally, the biomarkers and molecular mechanisms of the ABO blood group and AF recurrence were not thoroughly inspected in this retrospective study, which demands further investigations. The timeframe to examine the biomarker and the optimal cut-off values was uncertain [7]. The efficacy of biomarkers potentially changes over time, especially when the patient’s age and additional comorbidities influence those biomarkers leading to various results [7]. The serial inflammatory biomarkers before and after catheter ablation could be tested to identify the close relationship between the ABO blood group and inflammation. Regional cardiac inflammation should also be clarified between different blood groups.

## 6. Conclusions

The non-O-type blood groups (A, B, or AB type) were independent predictors for very late AF recurrence in the non-PAF patients after catheter ablation. The ABO blood group could be a useful biomarker for risk stratification of AF recurrence. The ABO blood groups in AF patients who underwent catheter ablation could help set up the pre-procedure treatment plan and trigger early awareness of AF recurrence in patients and physicians. This might change post-procedure care from actively detecting AF recurrence at an earlier stage to prompt diagnosis and therapy and hope to improve the long-term AF outcome.

## Figures and Tables

**Table 1:** Comparison of patient characteristics between the O-type and the non-O-type blood groups (A, B, or AB type).

Variables	O-Type Blood Group (*n* = 910)	Non-O-Type Groups (A, B, or AB Type) (*n* = 1196)	*p*-Value
Age	55.31 ± 11.43	55.93 ± 11.21	0.251
Men	678 (74.50%)	874 (73.13%)	0.461
HTN	389 (42.74%)	525 (43.94%)	0.598
DM	82 (9.03%)	142 (11.90%)	0.035
Dyslipidemia	184 (20.23%)	266 (22.24%)	0.262
Non-PAF	235 (25.82%)	314 (26.36%)	0.824
CHA_2_DS_2_-VASc Score	2.91 ± 1.02	3.02 ± 1.24	0.011
Amiodarone	172 (18.92%)	220 (18.44%)	0.767
Propafenone	47 (5.23%)	82 (6.91%)	0.109
Alcohol	194 (21.32%)	214 (17.92%)	0.049
Smoking	202 (22.21%)	268 (22.47%)	0.909
Echocardiogram			
LAD (mm)	38.20 ± 6.47	39.43 ± 6.74	0.007
LVEF (%)	58.65 ± 6.34	56.01 ± 7.33	0.044

AF = atrial fibrillation; DM = diabetes mellitus; HTN = hypertension; LAD = left atrial diameter; LVEF = left ventricular ejection fraction; PAF = paroxysmal atrial fibrillation.

**Table 2:** Comparison of patient characteristics with or without very late AF recurrence in the non-PAF patients after catheter ablation.

Variables	No Recurrence (*n* = 457)	Very Late Recurrence (*n* = 92)	*p*-Value
Age	54.52 ± 10.31	54.37 ± 10.12	0.863
Men	397 (86.92%)	77 (83.71%)	0.418
HTN	207 (45.32%)	39 (42.42%)	0.609
DM	52 (11.40%)	9 (9.81%)	0.657
Dyslipidemia	109 (23.94%)	21 (22.83%)	0.833
Non-O-Type Blood Groups (A, B, or AB type)	252 (55.17%)	62 (67.46%)	0.030
CHA_2_DS_2_-VASc Score	3.02 ± 1.18	3.12 ± 1.14	0.054
Amiodarone	227 (12.24%)	165 (66.33%)	<0.001
Propafenone	48 (2.67%)	81 (32.55%)	<0.001
Alcohol	109 (23.91%)	20 (21.77%)	0.663
Smoking	131 (28.73%)	22 (23.94%)	0.354
Echocardiogram			
LAD (mm)	42.13 ± 6.81	43.12 ± 7.44	0.217
LVEF (%)	58.6 ± 7.35	58.0 ± 7.34	0.233

**Table 3:** Univariate and multivariate analysis of very late recurrence in patients with the non-PAF.

Variables	Univariate Analysis		Multivariate Analysis	
	Odds Ratio (95% CI)	*p*-Value	Odds Ratio (95% CI)	*p*-Value
Age	0.99 (0.98–1.00)	0.021	0.99 (0.98–1.01)	0.260
Men	1.66 (1.19–2.33)	0.003	1.28 (0.88–1.86)	0.190
HTN	1.25 (0.96–1.62)	0.105	-	-
DM	1.17 (0.78–1.77)	0.442	-	-
Dyslipidemia	1.32 (0.97–1.79)	0.076	-	-
Non-O-type Blood Groups (A, B, or AB type)	1.33 (1.01–1.75)	0.039	1.40 (1.05–1.86)	0.022
CHA_2_DS_2_-VASc Score	1.01 (0.89–1.14)	0.919	-	-
Amiodarone	1.44 (1.08–1.93)	0.012	1.44 (1.08–1.93)	0.013
Propafenone	0.74 (0.53–1.02)	0.066	-	-
Alcohol	1.48 (1.09–2.02)	0.012	1.31 (0.91–1.89)	0.143
Smoking	1.67 (1.25–2.23)	0.001	1.22 (0.86–1.73)	0.257
Echocardiogram				
LAD (mm)	1.00 (0.98–1.02)	0.920	-	-
LVEF (%)	0.98 (0.96–1.00)	0.902	-	-

## Data Availability

The datasets generated during and/or analyzed during the current study are not publicly available due to confidential reasons but are available from the corresponding author upon reasonable request.
